# Pilot Study of the EncephaLog Smartphone Application for Gait Analysis

**DOI:** 10.3390/s19235179

**Published:** 2019-11-26

**Authors:** Keren Tchelet, Alit Stark-Inbar, Ziv Yekutieli

**Affiliations:** Montfort Brain Monitor LTD, Ha-Nasi 14, Zichron Ya’acov 3090314, Israel; keren@mon4t.com (K.T.); alit@mon4t.com (A.S.-I.)

**Keywords:** mHealth, timed up and go, iTUG, wearables

## Abstract

Gait disorders and falls are common in elders and in many clinical conditions, yet they are typically infrequently and subjectively evaluated, limiting prevention and intervention. Completion-time of the Timed-Up-and-Go (TUG) test is a well-accepted clinical biomarker for rating mobility and prediction of falls risk. Using smartphones’ integral accelerometers and gyroscopes, we already demonstrated that TUG completion-time can be accurately measured via a smartphone app. Here we present an extended app, EncephaLog^TM^, which provides gait analysis in much more detail, offering 9 additional gait biomarkers on top of the TUG completion-time. In this pilot, four healthy adults participated in a total of 32 TUG tests; simultaneously recorded by EncephaLog and motion sensor devices used in movement labs: motion capture cameras (MCC), pressure mat; and/or wearable sensors. Results show high agreement between EncephaLog biomarkers and those measured by the other devices. These preliminary results suggest that EncephaLog can provide an accurate, yet simpler, instrumented TUG (iTUG) platform than existing alternatives, offering a solution for clinics that cannot afford the cost or space required for a dedicated motion lab and for monitoring patients at their homes. Further research on a larger study population with pathologies is required to assess full validity.

## 1. Introduction

Abnormal gait patterns are common in a wide range of clinical populations, causing many discomforts and significantly increasing the risk of falls in seniors [1,2,3], neurological patients [4,5,6], and individuals suffering from orthopedic injuries [7,8]. Gait is commonly evaluated by the Timed Up and Go (TUG) test [9], which is usually conducted by a human rater, with TUG total completion time being the only quantitative outcome measured by a stopwatch. Scores for other gait components may also be subjectively evaluated by the rater, a method prone to intra- and inter-rater variability [10,11]. 

In order to provide quantitative and more detailed gait analysis, instrumented TUG (iTUG) has been introduced, using various devices for capturing a subject’s motion [12,13,14,15]. iTUG provides additional biomarkers that complement TUG completion time and augment the evaluation of disease progression [16,17]. On top of the TUG completion time, iTUG output includes the duration of TUG phases (Stand-Up, Walk-Away, Rotation, Walk-Back, and Sit-Down), as well as non-temporal parameters including step-related biomarkers and more [18,19,20,21,22,23,24,25]. iTUG’s higher accuracy in comparison to subjective evaluation has proven to be more sensitive to pathologies, useful in fall risk prediction [12,19,23,24,26,27], helpful in the evaluation of responses to therapeutic interventions [17,26], and can even provide cognitive related indicators [24,26,28].

Medical devices used for iTUG can be grouped into three main categories: (A) motion capture video cameras that record subjects’ gait from joint movements, (B) pressure sensor walkway arrays, that record walking pressures, and (C) wearables attached to the subjects’ bodies, that record sensor accelerations. All three technologies require dedicated and often expensive hardware, as well as trained professionals to operate the devices and extract the biomarkers. The first two groups also require devoted and large spaces to accommodate the hardware setup, making these solutions even more expensive and less accessible for most patients. 

EncephaLog^TM^ is a platform that utilizes smartphones’ internal motion sensors for conducting motor evaluation. We have already demonstrated the accuracy of EncephaLog’s TUG module (a.k.a AppTUG) in reporting TUG Completion Time in comparison to physicians’ reports [29]. In this paper we present how EncephaLog TUG can provide additional gait biomarkers on top of the TUG completion time, and how they match biomarkers obtained from representatives of the device categories mentioned above. Given the wide availability of smartphones, EncephaLog TUG offers an accessible and affordable iTUG solution that can take place at the clinic and/or in the patient’s natural environment. 

## 2. Materials and Methods

We chose one representative from each of the three categories of medical gait evaluation devices mentioned above, to be used as a reference to EncephaLog results. We performed a total of 32 TUG tests (×2 repetitions per test): 17 TUG tests were used for matching EncephaLog against motion capture cameras (MCC; Experiment 1), and 15 for matching against a walkway pressure mat and wearable sensors (tested in parallel; Experiments 2 and 3, respectively). All sessions were conducted on healthy subjects (*n* = 4). Data was collected simultaneously from the reference device and two smartphones running EncephaLog, an Android device (Galaxy S7 by Samsung Electronics, Suwon, South-Korea; 142.4 × 69.6 × 7.9 mm^3^, 152 g), and an iOS device (iPhone X, Apple, Cupertino, CA, USA; 143.6 × 70.9 × 7.7 mm^3^, 174 g). Smartphones were worn on the subjects’ body using a strap, a 5.5-inch phone waist bag commonly used for sport, on different body locations (Experiment 1: sternum, abdomen and hip; Experiments 2 and 3: sternum).

**Figure 1:**
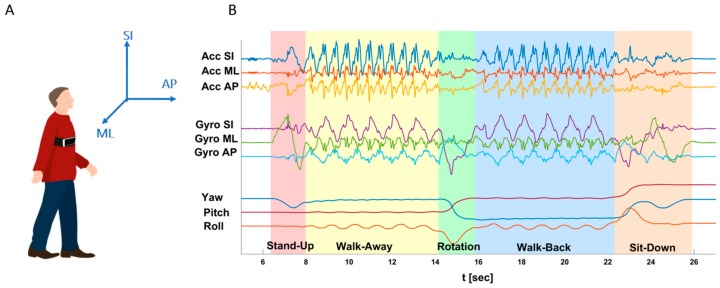
Timed-Up-and-Go (TUG) conventions. (**A**) Human coordinate system. Anterior-Posterior (AP) is the walking direction. (**B**) Event detection in EncephaLog TUG. Example of EncephaLog data from one TUG test, including nine raw signals collected by a smartphone device (lines), and the five TUG phases detected offline (shaded backgrounds). See Table A1 for events and biomarkers definitions.

All data was analyzed offline, using Python 3.6.0 and JMP 14 (SAS institute). Signals from all four methods were aligned to the subject’s physical space (Superior-Inferior (SI), Anterior-Posterior (AP), and Medial-Lateral (ML); Figure 1A), to put the data in a meaningful physiological context and to use a unified coordinate system. Five TUG phases were identified (Figure 1B, shaded backgrounds): Stand-Up (SU), Walk-Away (WA), Rotation (Rot), Walk-Back (WB), and Sit-Down (SD) [14,24,30,31,32], and up to 10 biomarkers were extracted and compared between methods (within specific system limitations, see below) including six temporal biomarkers: Completion-Time (CT; the traditional TUG biomarker), Stand-Up Time, Walk-Away Time, Rotation Time, Walk-Back Time, Sit-Down Time; and four non-temporal biomarkers: number of Steps (from straight walking phases), number of Rotation Steps, average Steps Frequency (Cadence), and average Steps Length. Note that in some cases, the reference devices were limited in their ability to capture some phases of the TUG protocol, e.g., the walkway arrays cannot properly track the Stand-up and Sit-down patterns. These sections were therefore excluded from comparison.

We tested the agreement between EncephaLog and reference devices, looking at the concordance in measuring the same biomarkers by the various methods, given that all motion sensors aim to measure the same biomarkers. For that, we calculated the difference between each of the 10 biomarkers extracted from EncephaLog and the equivalent biomarker from each other device, using Bland–Altman (B&A) statistical method [33]. For each pair Encephalog and one of the three reference devices, the mean of differences $\bar{d}$ between methods, and the maximum relative error (Equation (1)) were obtained over all samples. In cases of correlation between the differences and the averages of two methods, B&A analysis was applied after log transformation. A two-tailed *t*-test was used to compare the difference between methods to zero (per biomarker), in order to test whether there was a systematically fixed bias (i.e., no agreement) between methods. We further report upper and lower 95% confidence intervals of the mean differences between methods, per biomarker. If the differences are within the confidence intervals, they are not clinically important, indicating that the two methods may be used interchangeably. 

(1) $$\;\;\%error=\frac{abs\left(golden\;standard-EncephaLog\right)}{golden\;standard}\cdot 100\%.$$

In Experiment 3, we further calculated the cross correlation (R) and the goodness of fit using normalized root mean square error (NRMSE) between raw EncephaLog signals and wearable sensors signals, expecting high and low values respectively.

### 2.1. EncephaLog

EncephaLog^TM^, by Montfort Brain Monitor (Zichron Ya’acov, Israel), is a smartphone-based platform for motor and cognitive evaluation. One of the test protocols supported by EncephaLog is the TUG test. The test starts with the subject sitting on a chair, and the smartphone attached to his/her body by means of a strap. Once the subject is ready for the test, indicated by pressing a “begin” button, an auditory countdown is heard, followed by auditory and vibratory “start” signals. The subject then stands up from a chair, walks a few meters, turns, walks back to the chair, and sits back down. During the test, EncephaLog uses three accelerometers (Acc; Figure 1B, top three lines) and three gyroscopes (Gyro; Figure 1B, middle three lines) to measure subjects’ motion patterns. Furthermore, smartphones provide three phone orientation signals (Yaw, Pitch, Roll; Figure 1B, bottom three lines) derived from the six sensors. EncephaLog TUG validates the proper execution of the test, and when the test is completed, it accurately reports the TUG Completion Time [29]. The raw sensors’ data is transmitted to the cloud (Microsoft Azure Storage), resampled to 100 Hz, followed by offline signal processing and semi-automatic TUG phase detection and biomarker extraction. Appendix Table A1 describes the definition of the EncephaLog phases and biomarkers. 

Many previous studies using wearable sensors for gait and posture recording place the device on the body’s center of mass (COM), that is on the horizontal plane of the navel, and usually on the lower back (and not on the abdomen) [11,28,34]. In preliminary studies we found that the location most suitable for measuring the ML Sway biomarker (not presented in this paper) is the sternum, as every degree of COM tilt is manifested in a larger distance shift of the sternum than of the COM itself, magnifying sway amplitude. For all other biomarkers, we did not find a significant effect of smartphone location. In order to artificially increase data variance, we placed the smartphones on multiple body positions (sternum, abdomen, and on hip) during Experiment 1. 

### 2.2. Motion Capture Cameras (MCC)

**Figure 2:**
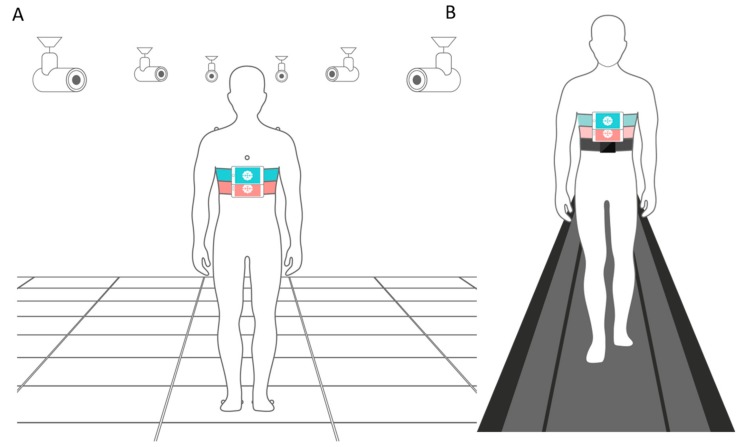
TUG experimental setups. (**A**) Experiment 1. The motion capture cameras (MCC) setup included a system of 10 (six drawn for simplicity) motion capture cameras, and eight passive infrared sensors placed on the subject’s body (only seven displayed since the eighth was located on the subject’s back). Additionally, two smartphones devices (Android and iPhone) running EncephaLog were strapped to the subject’s sternum. (**B**) Experiments 2 and 3. Two smartphones devices (Android and iPhone) running EncephaLog and one wearable sensor (Opal) were strapped to the subject’s sternum, while he/she walked on a pressure walkway mat.

A system of video-based motion capture cameras (6+ series by Qualisys™, Göteborg, Sweden [35]) was used for TUG recording, and Visual 3D V6 software (C-Motion, Germantown, MD, USA) was used for offline gait analysis. This video-based method offers high performance, precision, and usability in biomechanics, using 10 motion capture cameras and eight passive infrared reflective markers on the subject’s body (four for upper body: sternum, back, and each shoulder; and two for each foot; see Figure 2A). Each marker provides 3D coordinates over time. Using a special calibration kit, an experienced technician calibrated the cameras system before the experiment. Given the cameras’ fields of view visibility limitation [36], only 5 m were visible enough for the TUG test (out of an 8 m walkway space) and therefore tests were limited to that distance. 

EncephaLog tests (*n* = 17) were completed by one subject (43 year-old male), during Experiment 1, using three different device locations (sternum, abdomen, or hip); two walking distances (5 m, the longest walking distance available in the MCC lab; and 3 m, the common TUG distance); and various walking patterns (different speed, different step length, etc.) In each test, the subject performed two TUG trials and their output biomarkers were averaged.

**Figure A1:**
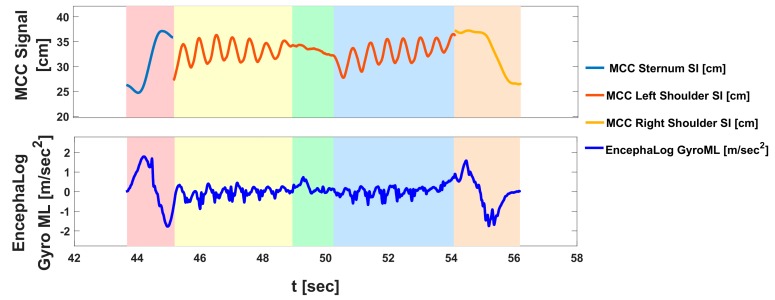
MCC data analysis. MCC signals are composed from multiple sensors. Top: An example of MCC “SI signal” composed from data of 3 infrared markers (sternum SI, left shoulder SI, right shoulder SI, marked in cyan, red, and orange lines, respectively) combined in order to obtain one full SI signal that was used in this study. Bottom: Equivalent data from EncephaLog is captured by a single sensor (gyro ML, marked in blue). Shaded backgrounds represent the five TUG phases detected offline, as in Figure 1.

The output of the MCC system is coordinates in 3D space over time, per marker, without video frames. The combination of limited cameras’ fields of view and the biological movement during the TUG test create “missing” data, as not all infrared reflective markers are continuously captured throughout the test. The main challenge of TUG data analysis via this method is therefore to find which markers provide the data required for each gait biomarker and how to synchronize between markers and signals, or between markers/signals and gait patterns (see examples in Figure A1). Therefore, experts working in the video lab conducted this initial analysis with Visual 3D software, synchronizing markers signals in time. We then plotted those signals to identify TUG phases according to physical 3D movement trajectories in space and time.

### 2.3. Pressure Mat

In Experiment 2, a Zeno™ Walkway pressure mat and PKMAS gait analysis software (by ProtoKinetics, Havertown, PA, USA; [37,38]) were used, consisting of a 40 × 800 cm^2^ pressure sensing carpet, measuring body pressure data during walking. Pressure coordinates were recorded during walking, mapping step patterns from which the Center of Pressure (COP) trajectory was calculated, along with step cadence and step length. The Center of Mass (COM) trajectory can be extrapolated from the COP, based on the correlation between the two parameters (COM, COP) [32,39,40] but the extrapolation process may introduce errors and yield an inaccurate COM parameter. 

Four healthy adults (three males and one female of age 33.5 ± 3.9) participated in 30 TUG trials (*n* = 15 tests, two trials per test) of 8 m, with the smartphones strapped to the sternum (Figure 2B) during all trials, and while involving different walking patterns (as in Experiment 1). 

Raw data from the pressure mat contains time stamps of the pressure (force) produced by the subjects’ feet. Given that the durations of the TUG phases do not depend on feet contact with the mat, TUG temporal biomarkers could not be extracted from this system. Without the information pairing specific steps to the beginning/end of each phase, it is very difficult to detect which steps refer to each phase (e.g., to differentiate between SU and WA phases). The inability to place a chair on the pressure mat makes it impossible to measure the duration of the SU and SD phases. The mat is also not sensitive enough to properly identify the Rot phase because the output force signals in this short event are performed over a small area of the mat, making it difficult to detect separate steps as well as accurately identify the start and end points of the rotation movement. Altogether, only four of the 10 biomarkers extracted from EncephaLog were also extracted from the pressure mat. It is important to mention that the pressure mat can provide other biomarkers that cannot be extracted from EncephaLog, like toe angle in each step, etc. [11,38].

### 2.4. Wearable Sensors 

The Opal™ system (by APDM, Portland, OR, USA [41] consists of wireless movement sensors to record and store data of functional mobility, including gait and balance. The system uses Micro Electro Mechanical Systems (MEMS) technology to record movement with triaxial accelerometers, gyroscopes, and magnetometers. The system wirelessly synchronizes simultaneous sensors. One Opal wearable sensor (WS; sampling rate of 128 Hz) was positioned on the subjects’ sternums, alongside the two smartphones. The procedure of Experiment 3 was the same as that of Experiment 2 (see Figure 2B). 

We conducted two types of comparisons between EncephaLog and Opal WS data. First, given that both methods are based on the same types of sensors, we compared the raw data (from three accelerometers and three gyroscopes) between the two methods using cross-correlation and NRMSE. Signal pre-processing included the detection of matching axes from the two methods based on the similarity of the signal’s general behavior, followed by several signal processing steps. First, we temporally aligned the raw signals based on the torso’s first bending forward movement during the Stand-Up phase, as detected by the gyro ML signal. Second, we resampled both signals to 103 Hz. Finally, we reduced the bias/DC components. After these steps, there were still differences between the signals in the SI direction (especially during the SU and SD phases), presumably due to different handling of the gravitation component in the different devices. 

Following pre-processing, data from both resources was processed through the same processing pipeline (by synthetically entering WSs data into EncephaLog’s CSV data format) to extract the same biomarkers. We then tested the agreement between WSs and EncephaLog’s produced biomarkers, as was done for the two other methods. 

## 3. Results

Table 1 and Table 2 summarize the comparison between biomarkers extracted from EncephaLog and biomarkers extracted from each of the three standard motion sensors used in this study. We obtained the mean of differences (Table 1) and the maximum relative error (Table 2) between the two methods for Android (top row) and iPhone (bottom row) devices separately. All temporal biomarkers show mean differences lower than 1.045 s. The maximal relative errors are not higher than 33% and 13% for the MCC and WS methods, respectively (note that the pressure mat does not provide temporal biomarkers). It is not surprising that EncephaLog shows higher agreement with the WS than with the MCC, as both EncephaLog and the WS uses a similar type of sensors (accelerometers and gyroscopes), while the MCC is based on joint position in space. 

The maximal mean over all step related biomarkers is 2.5 steps, and the maximal relative errors found are in the range of 50% (MCC) and 20% (WS and pressure mat). We assume that the larger maximal relative errors of the steps related parameters as opposed to the temporal parameters (from the same devices), result from intra rater biases, which are larger for the steps related parameters. A common disagreement can be influenced by the decision of which steps to include in the Rotation phase vs. which steps to relate to straight walking phases. A difference of even one single step, out of 3–4 steps typical to the Rotation phase or out of 6–8 steps typical to the WA phase, strongly affects the step biomarkers, and thus yields high relative errors. Moreover, this difference is enlarged by the fact that different raters and algorithms extracted the biomarkers from MCC and EncephaLog. 

Specifically, Step Frequency and Step Length biomarkers showed high agreements between EncephaLog and each of the three motion sensors methods used: maximal mean difference of 0.656 steps/s and 0.036 m, respectively. The relative errors of Pressure walkway and WS were lower than 14.2% but for MCC there were maximal errors of 49%, pointing to disagreement between EncephaLog and MCC, and highlighting the need for additional experiments with more subjects and samples.

Overall, EncephaLog and Opal WS were most similar; an expected results given that both measure movement accelerations (linear and angular) taken from the same position (sternum). These results add to those we previously showed, that EncephaLog provides accurate measurements of the standard TUG biomarker reported by neurologists using a stopwatch, the total Completion Time (where the EncephaLog – neurologists’ correlation coefficient was R > 0.99 per rater [29]).

### 3.1. Experiment 1: EncephaLog vs. MCC

**Figure 3:**
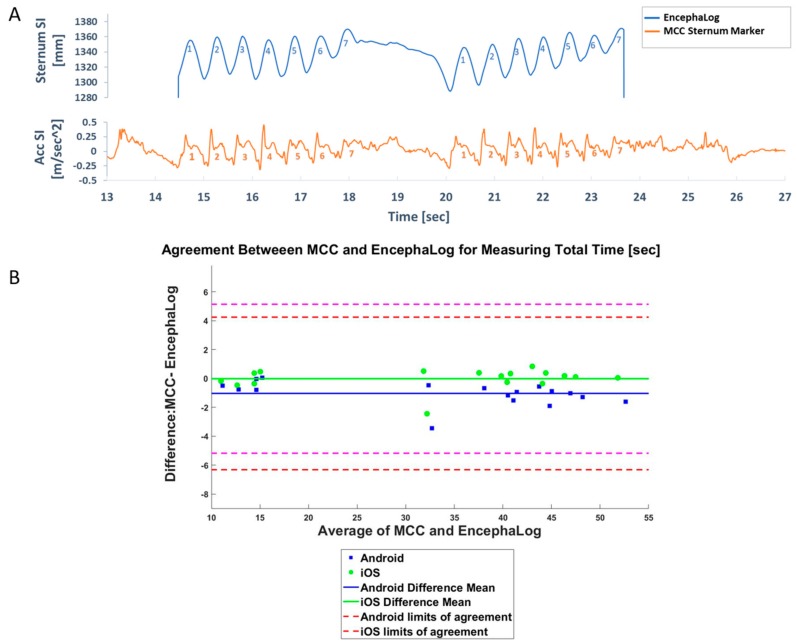
MCC results. (**A**) Example of a visual comparison of MCC (top) and EncephaLog (bottom) signals. Note the similarity in the number and duration of steps during TUG Walk Away (WA) and Walk Back phases (WB), despite the different recourses: Superior-Inferior (SI) coordinates in 3D space in the MCC, and acceleration-SI in EncephaLog. (**B**) Bland–Altman plot, reflecting the degree of agreement between MCC and EncephaLog for TUG Completion Time (*n* = 17 samples). Presented here is data from an Android (blue) and iOS (green) devices. Dashed lines represent 95% confidence intervals of Bland–Altman (B&A) for Android (magenta) and iOS (red). Similar results and graphs were obtained for all biomarkers in this experiment.

In this experiment, one subject performed *n* = 17 TUG tests, with different smartphone locations. Three trials were not properly recorded by the MCC system and therefore only a single repetition was analyzed for those tests, as opposed to the average over two repetitions (trials) for all other tests. Signals from the two methods resemble each other (Figure 3A), and the highest agreement was seen for the Completion Time, where the maximal relative error was 11.192%. This biomarker is the only quantitative outcome in conventional TUG, classically measured by a stopwatch (Figure 3B). Note that the results were almost the same regardless of smartphone position.

### 3.2. Experiment 2: EncephaLog vs. Pressure Mat

**Figure 4:**
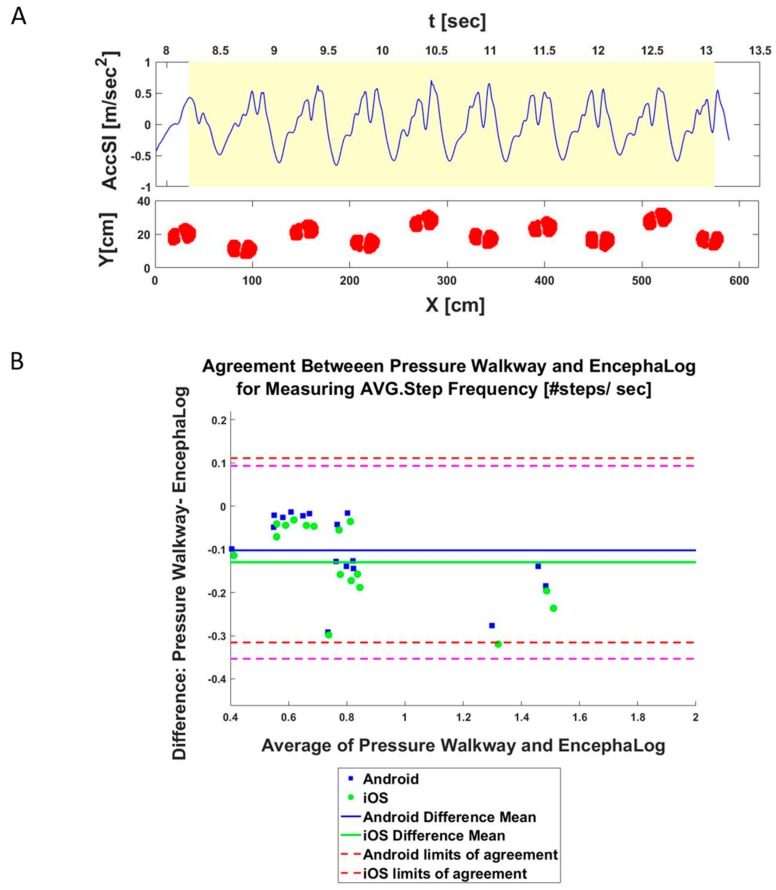
Pressure mat results. (**A**) Example of a visual comparison of pressure mat (bottom) and EncephaLog signals (top). Note the similar walking patterns captured simultaneously by both methods. (**B**) B&A plot, an agreement measurement for Average Steps Frequency between pressure mat and EncephaLog (*n* = 15 samples), performed on Android (blue) and iOS (green) devices.

In this experiment, four subjects performed *n* = 15 TUG tests, all with with the smartphones placed on the sternum. Out of the 10 biomarkers EncephaLog can extract, the pressure mat can extract only four biomarkers, as it contains temporal information only for the walking sections and is not sensitive enough to properly identify the rotation phases (see Materials and Methods). Despite the different types of data, similar walking patterns were captured by both methods (Figure 4A). Mean differences between EncephaLog and pressure mat biomarkers are small, with correspondingly small relative errors. The lowest maximal relative error between the two methods was seen for Step Frequency (i.e., Cadence; Figure 4B), which is one of the most important gait parameters, used as an indicator for neurological classification and disease progression [1,12,42].

### 3.3. Experiment 3: EncephaLog vs. Other Wearable Sensors

**Figure 5:**
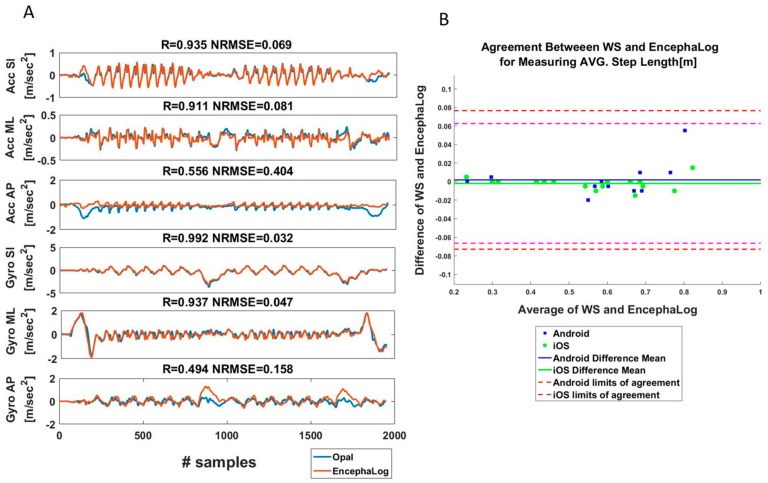
Wearable sensor results. (**A**) Comparison between EncephaLog and Opal WS of linear and angular accelerations. Correlation coefficient (R) and Normalized Root Mean Square Error (NRMSE) between signals of the two methods are presented above each graph. (**B**) B&A plot, an agreement measurement of Average Step Length between Opal and EncephaLog (*n* = 15 samples) performed on Android (blue) and iOS (green) devices.

**Figure A2:**
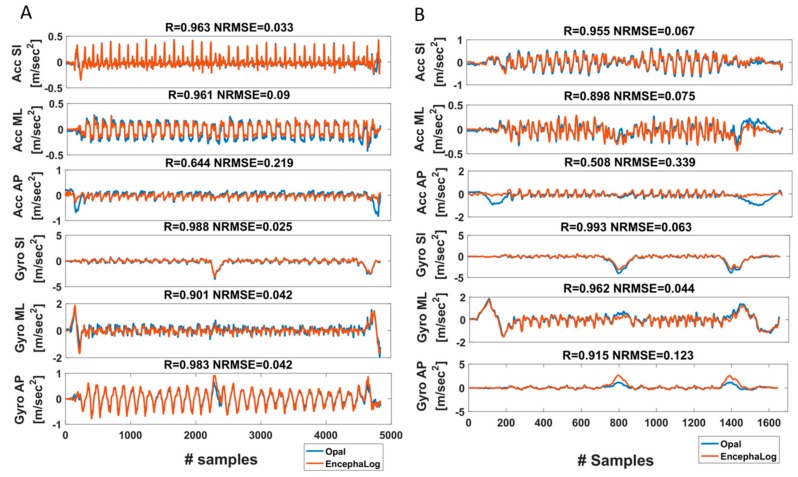
Examples for Opal (**a**) vs. EncephaLog (**b**) Raw Signals Correlation. Results from two different walking patterns.

In the third experiment, we first compared the preprocessed accelerometer and gyroscope signals (three axes each; see Materials and Methods) between the smartphones and ‘Opal’ WS (per trial; *n* = 30). This analysis can be conducted only in this experiment given the similarity between the signals. As can be seen in the example presented in Figure 5A (from one representative test; see additional examples in Figure A2), most of the signals highly overlap, except for some short intervals such as the SU or SD phases in Acc AP signal, or Rot in Gyro AP, overall leading to high agreements between biomarkers (Figure 5B). We assume that these differences emerge from different filtering processes of the gravity in the SI direction between the sensors of EncephaLog and Opal. For example, during SU, subjects bend their torso forward, causing a large change in sternum position along the AP direction, evident by large Acc AP and Gyro ML signals. As a result, in some trials the correlations between the signals are low and the normalized root mean square error (NRMSE) is high, leading to unsatisfying average values (Table 3) of AccAP and GyroML (R = 0.568 and NRMSE = 0.271) relative to the other signals (R > 0.86 and NRMSE < 0.13 for AccSI, AccML, GyroSI, GyroAP). 

Overall, as presented in Table 1 and Table 2, there is high agreement between EncephaLog and ‘Opal’ biomarkers, expressed by only small mean differences between the two methods for all biomarkers, and a maximal relative error of 20% for # Rotation Steps, reflecting substantial similarity between EncephaLog (Android and iOS) and Opal wearable sensors.

## 4. Discussion 

This pilot study demonstrated preliminary results for validating EncephaLog, a new smartphone application for gait analysis, against standard medical devices used in movement labs that can be used for iTUG. Our results show that EncephaLog can provide accurate biomarkers as other, more expensive and complicated, methods. On top of the gold standard Completion Time, EncephaLog provides nine additional biomarkers. All 10 quantitative biomarkers are much easier and faster to obtain from EncephaLog, than from representative technologies, which are not necessarily dedicated for TUG. With the rising popularity and ubiquitous usage of smartphones [43], EncephaLog is expected to provide a more accessible, and therefore scalable, tool for TUG-based gait analysis.

### 4.1. EncephaLog vs. Other Methods 

In a previous study [29] we showed that EncephaLog Completion Time and conventional TUG Completion Time evaluated by experienced movement disorder neurologists, highly correlate (R = 0.982 and R = 0.999 for *n* = 50 normal pressure hydrocephalous (NPH) and *n* = 50 healthy controls samples, respectively). Here we expanded the scope of the previous study, by comparing the agreement between EncephaLog and three other conventional gait measuring technologies for 10 TUG biomarkers in a small sample of subjects. 

We found that despite the high sensitivity and accuracy of motion capture cameras [44], this technology is not ideal for TUG, given the cameras’ restricted field view, preventing simultaneous capture of all body sensors throughout all TUG phases. In a previous study using the Qualisys™ MCC, subjects repeated the TUG test six times, prioritizing different TUG phases during distinctive test repeats, and while wearing approximately 50 body markers [45]. Despite the robust procedure, the physical field of views of the cameras made it impossible to analyze biomarkers such as the complete gait cycle [45]. MCC further require a large physical space to house the system, cumbersome procedures (e.g. attaching multiple body sensors [11,45]) and professionals to calibrate and operate the system, analyze the data, and extract the TUG biomarkers; altogether making MCC expensive, time consuming, and less accessible for iTUG. 

Similarly, pressure mats also require large designated spaces [11,45]. These systems mainly focus on straight walking and cannot provide Stand-Up and Sit-Down information, limiting the ability of pressure mats in measuring TUG. 

The last experiment showed that raw data from smartphone sensors is very similar to raw data from designated movement tracking body sensors [46], which were used in a previous study and demonstrated high within-session reliability over three iTUG repetitions [47]. The simultaneous Opal and EncephaLog measurements we report here are more strongly correlated than the within-session Opal correlations previously reported [47], reflecting the synchrony of the measurements despite being acquired from different systems, which in turn highlights the accuracy of EncephaLog relative to other commercially available wearable systems. The main advantages of EncephaLog over other wearable systems is that EncephaLog does not require neither dedicated and expensive wearable sensors, nor separate software to run the TUG test and/or to see the results, thus allowing the test to be performed in any neurological clinic as well as in the community. 

### 4.2. EncephaLog Benefits for iTUG

Despite TUG’s popularity, the conventional test faces a few drawbacks that EncephaLog addresses. First, EncephaLog provides an objective assessment of the subjectively measured gold standard outcome of the test, Completion Time. EncephaLog simultaneously records nine signals from the beginning of the countdown, throughout the test, till a few seconds after sitting down on the chair. Following the initiation of the test, all recordings are automatic, including the decision when the test is completed, and when to terminate the recording (when all accelerometers are below a predefined threshold for a couple of seconds). Thus, EncephaLog eliminates any subjective decisions and therefore improves the quality of the data in general, and of the completion time in particular. 

EncephaLog applies a fixed TUG protocol to address these inconsistencies, and its large (and growing) normed Big Data database allows clinicians to compare a new patient to a subset of subjects with similar age, gender, or disease, or to compare repeating tests from the same subject over time in order to monitor disease progression. 

Third, EncephaLog provides nine additional biomarkers to the gold standard TUG outcome, objectively and quantitatively expanding the clinical coverage of gait biomarkers. Rotation time and step length provide information on medication-induced dyskinesia that worsens PD patients’ gait [26,48] and are used for assessing PD progression (characterized by slower rotation and smaller step length [12]). Elders and those with Parkinson’s disease who fall have a tendency toward a lower cadence, especially when combined with shorter stride [49,50]. Therefore cadence is used as a quantifying measure for disease monitoring and rehabilitation as well as the assessment of fall risk [1]. It is therefore important to measure multiple biomarkers to monitor disease status and treatment side effects, to gain a more complete clinical gait picture. 

### 4.3. Study Limitations 

This study was conducted on a small number of healthy subjects. In order for EncephaLog to replace the traditional and standard TUG method in clinical settings, we must validate it on typical clinical populations, not only on healthy controls. Moreover, while EncephaLog accurately provides multiple biomarkers, it does not provide all possible iTUG-related biomarkers. The other methods against which we compared EncephaLog biomarkers can provide other important biomarkers, such as toe angle, Plantar/Dorsi flexion, etc. [11,24,51,52] We are currently developing algorithms to analyze such additional biomarkers, that will be presented in future work. 

## 5. Conclusions

This study presents that by using the EncephaLog app, a standard smartphone can be used for providing quantitative TUG analysis which includes temporal and step-related biomarkers. The results from this study show promising preliminary validation results on a pilot dataset, and warrant further validation work on larger patient populations. The results also point to the advantage of having an accessible iTUG solution that does not require a dedicated laboratory and equipment, as well as being able to conduct the test and extract the results by anyone, rather than by trained medical personnel.

## Figures and Tables

**Table 1:** Bland–Altman agreement results between EncephaLog biomarkers and biomarkers from each of the three technologies used (top row—Android, bottom row—iOS) across all trials, including Mean of differences ($\bar{d}$), confidence intervals (95%), and the *p* value for two tailed *t*-test comparing the differences to zero. (-) Marks biomarkers that could not be derived from the pressure mat. All confidence intervals include 0, and all *p*-values > 0.05, indicating that we cannot reject H0, therefore we can state that the biomarkers are similar and there is a fine agreement between EncephaLog and each of the three methods.

	Motion Capture Cameras *n* = 17			Pressure Mat *n* = 15			Wearable Sensors *n* = 15		
	$\bar{d}$	Agreement Limits	*p*-Value	$\bar{d}$	Agreement Limits	*p*-Value	$\bar{d}$	Agreement Limits	*p*-Value
**Completion Time (s)**	−1.044	[−6.327,4.238]	0.833	-	-	-	−0.858	[−4.938,3.222]	0.409
	−0.026	[−5.184,5.132]	0.996	-	-	-	0.039	[−3.945,4.024]	0.496
**Stand-Up Time (s)**	−0.024	[−0.903,0854]	0.855	-	-	-	−0.051	[−0.350,0.249]	0.299
	0.031	[−0.848,0.910	0.812	-	-	-	−0.003	[−0.305,0.298]	0.485
**Walk-Away Time (s)**	−0.350	[−3.568,2.868]	0.874	-	-	-	−0.307	[−2.543,1.928]	0.424
	0.018	[−3.144,3.179]	0.994	-	-	-	0.003	[−2.193,2.198]	0.424
**Rotation Time (s)**	0.319	[−0.908,1.545]	0.481	-	-	-	−0.082	[−0.533,0.369]	0.308
	0.401	[−0.801,1.603]	0.369	-	-	-	0.005	[−0.481,0.490]	0.488
**Walk-Back Time (s)**	−0.970	[−3.706,1.766]	0.656	-	-	-	−0.286	[−2.147,1.574]	0.429
	−0.532	[−3.146,2.083]	0.804	-	-	-	0.026	[−1.730,1.781]	0.499
**Sit-Down Time (s)**	−0.014	[−1.414,1.387]	0.962	-	-	-	−0.038	[−0.899,0.823]	0.422
	0.061	[−1.279,1.402]	0.828	-	-	-	0.082	[−0.678,0.842]	0.334
**Rotation Steps (#)**	1.912	[−1.200,5.024]	0.243	-	-	-	0.031	[−0.911,0.974]	0.407
	1.912	[−1.200,5.024]	0.243	-	-	-	0.000	[−0.943,0.943]	0.500
**Walking Steps (#)**	−2.454	[−9.771,4.864]	0.633	−1.103	[−6.301,4.096]	0.637	−0.092	[−5.646,5.462]	0.484
	−2.485	[−9.723,4.753]	0.628	−1.035	[−6.255,4.185]	0.665	−0.025	[−5.431,5.381]	0.496
**AVG. Steps Frequency (Cadence) (#/s)**	−0.102	[−0.316,0.111]	0.352	0.030	[−0.026,0.086]	0.691	0.026	[−0.028,0.079]	0.367
	−0.130	[−0.354,0.093]	0.381	−0.001	[−0.026,0.086]	0.958	−0.003	[−0.062,0.055]	0.483
**AVG. Steps Length (m)**	−0.015	[−0.087,0.058]	0.849	0.036	[−0.049,0.121]	0.556	0.002	[−0.073,0.077]	0.488
	−0.015	[−0.086,0.057]	0.849	0.033	[−0.052,0.117]	0.596	−0.002	[−0.066,0.063]	0.488

**Table 2:** Maximum relative error % between EncephaLog biomarkers and biomarkers from each of the three technologies used (top row—Android, bottom row—iOS). (-) Marks biomarkers that could not be derived from the pressure mat.

	Motion Capture Cameras *n* = 17	Pressure Mat *n* = 15	Wearable Sensors *n* = 15
**Completion Time (s)**	11.192	-	4.959
	7.930	-	2.737
**Stand-Up Time (s)**	21.324	-	7.957
	20.956	-	5.263
**Walk-Away Time (s)**	21.313	-	6.381
	16.794	-	1.725
**Rotation Time (s)**	34.112	-	13.302
	37.617	-	6.400
**Walk-Back Time (s)**	32.904	-	3.896
	28.860	-	3.506
**Sit-Down Time (s)**	22.203	-	10.961
	15.734	-	8.511
**Rotation Steps (#)**	50.000	-	20.000
	50.000	-	0.000
**Walking Steps (#)**	40.450	22.124	18.771
	41.466	16.628	2.258
**AVG. Steps Frequency (Cadence) (#/s)**	49.564	7.204	4.996
	50.646	3.874	2.500
**AVG. Steps Length (m)**	19.586	13.308	6.627
	17.347	14.159	2.256

**Table 3:** Experiment 3—correlation of raw signals between EncephaLog and ‘OPAL’ wearable sensors. Average cross-correlation coefficients (R) and normalized root mean squared error (NRMSE) between sensors signals *(n* = 30 trials).

	Acc SI(X)		Acc ML(Y)		Acc AP(Z)		Gyro SI(X)		Gyro ML(Y)		Gyro AP(Z)	
	R	NRMSE	R	NRMSE	R	NRMSE	R	NRMSE	R	NRMSE	R	NRMSE
**AVG**	0.907	0.062	0.881	0.13	0.568	0.271	0.861	0.071	0.787	0.068	0.875	0.069
**STD**	0.14	0.049	0.121	0.082	0.097	0.058	0.225	0.07	0.243	0.046	0.208	0.069

## Appendix A

**Table A1:** EncephaLog biomarkers and events definitions.

Completion Time (s)	Completion time of entire TUG sequence. Taken from the "Go" command until the subject re-sits with his/her back leaning on the chair’s backrest, identified by flattening of all signals (mostly identified by the Gyro ML signal).
Stand-Up time (s)	Begins when the subject stands up until the first step forward. Mostly identified from the Gyro-ML signal, which dramatically increases during the initial bending forward of the torso during stand-up, and then decreases dramatically at the straightening of the torso and return of the signal to baseline.
Walk-Away time (s)	From the first step forward (end of SU phase) until the first rotation step. During this phase, all accelerometers and gyroscopes are characterized by a harmonic, cyclic pattern, while the Yaw, Pitch, and Roll are flat.
Rotation Time (s)	When the subject turns around, until the first walking backward step. Clearly evident by a 180 degrees Yaw shift. Gyros SI and AP also change dramatically, while the accelerometers become quite flat, lacking the rhythmic pattern of straight walking.
Walk-Back Time (s)	Between rotations – from the first step backward after the end of the Rotation phase, until the last second before the turning part of the Sit-Down phase. Similarly to the WA phase, characterized by harmonic accelerometers and gyroscopes pattern, while the Yaw, Pitch, and Roll are flat.
Sit-Down Time [s]	Incudes a turn to orient the body to the chair and sitting down on the chair. Starts with the first turning step, indicating the intention to sit down, until the subject sits "fully" on chair with his/her back leaning on the chair’s backrest. As in the Rotation phase, the Yaw changes dramatically from 180 degrees back towards 0, and the Gyros perform a dramatic change.
Walking Steps (#)	Average number of steps during the straight walking phases (WA and WB; excluding steps while rotating).
Rotation Steps (#)	Number of steps during the Rotation phase.
AVG. Steps Frequency (Cadence) (#/s)	Cadence, i.e. the AVG. number of steps per second, during straight walking (WA and WB).
AVG. Steps Length (m)	Average of step length, defined as one heel strike followed by the other heel strike (half a cycle). Taken from straight walking phases (WA and WB) only.

### Motion Capture Cameras (MCC) Data Analysis

Signal extraction: As mentioned in the Materials and Methods section, some infrared markers are blocked from the cameras’ view during some TUG movements, requiring the combination of signals from multiple markers in order to obtain a full signal required for the biomarker’s extraction process. Experts working in the MCC lab manually conducted this initial analysis with Visual 3D software. For example, when standing up, the upper body bends forward, hiding the sternum marker for 1-2 sec. To overcome missing data, data from 3 other markers (back, left and right shoulder) needs to be combined (see example Figure A1). For all steps-related parameters (Steps Average Length, Steps Cadence (frequency), Number of Steps), signals were combined from three markers: Sternum SI and 2 feet markers (one from each foot) in AP direction.
